# Medical and Obstetrical Cases

**Published:** 1847-03

**Authors:** R. L. Scruggs

**Affiliations:** Germantown, Tennessee


Art. II.—*Medical and Obstetrical Cases.* By R. L. Scruggs, M. D., of Germantown, Tennessee.

Pneumonia. May 1st, 1846. Visited G. F., aged about 23 years, in Upper Mississippi; found him delirious, having been attacked with pneumonia of both lungs four days previously, for which little or nothing had been done. His pulse being 120 in the minute and tolerably full, skin dry and hot, and head much affected, the result of want of due arterialization of the blood, I determined to use the lancet. This I did cautiously, and finding, after I had taken a few ounces of blood that he appeared to sink under its loss, I closed the orifice. I then cupped him extensively over the chest, and applied mustard mixed with hot vinegar over the chest, and upper and lower extremities, which made not the slightest impression. I then mixed together equal parts of hot oil of turpentine and vinegar and rubbed the mixture upon the extremities and chest; immediately after which I applied sinapisms to the extremities and a large blister over each lung. These having made an impression very soon, the sinapisms were removed and the blisters permitted to remain until they drew well; after which, for several days, the blistered surfaces were dressed with savin cerate, and whenever they showed a disposition to heal the blisters were reapplied. I gave him a solution of mur. ammon. and tart. emetic every hour, in teaspoonful doses, to be increased or diminished according to the effect produced upon the stomach. On the 3d day after I first saw him I gave an expectorant of lobelia, squills, and seneca; gave blue pill grs. v., night and morning from the commencement, and continued it until its constitutional effects were discoverable. This, with a little Dover's powder to prevent the medicine from running off by the bowels, and warm teas to promote diaphoresis, with abstemious diet, &c., constituted the treatment.

This patient had been delirious for fifty-six hours before I saw him, and remained so for twelve hours afterwards. On my first examination I found the lungs filled from their bases to very near their apices; the patient's breathing was very rapid, of course, and that dull redness of the cheeks peculiar, I believe to this disease—the effect of venous congestion, or a languid circulation in the capillaries—conspicuous at first sight. He soon recovered, notwithstanding all the unfavorable appearances, and is now in perfect health.

Rupture of the Uterus. Visited Mrs. B., in her fifth or sixth labor; found her with a dull, inexpressive, and sunken eye; countenance anxious and dejected; abdomen so exceedingly tender that she could not bear to have it touched. I found an irregular practitioner in attendance, with several old women, who informed me that the patient had had occasional pains, resembling labor pains for three weeks; but that she had been in actual labor for three days; that the head had been the presenting part originally, but that they had attempted to "push it up and turn," in which efforts they had succeeded in getting hold of one hand of the fœtus, at which they had "pulled with all their strength without being able to move it an inch;" that the pains had been strong up to 12 o'clock the night before, at which time they had ceased altogether. Upon making an examination per vaginum, it was found that the child had receded entirely out of the reach of the finger. The man in attendance, told me that he had given large and repeated doses of ergot, and pulled hard at the *hand* of the fœtus during the violent contractions of the uterus brought on by the ergot.

It was clear to my mind that the uterus was extensively ruptured, and that the poor woman had been sacrificed by the ignorance of those who attended her. Notwithstanding the pulse was quick, irregular and very feeble, and the respiration hurried, labored and painful, I determined to turn and deliver, believing it to be the only possible hope. I gave her a large dose of morphia, from the effects of which she slept about three quarters of an hour. Immediately after she awoke, I passed my hand into the peritoneal cavity, without feeling the uterus, and with some difficulty disentangled the fœtus from the intestines and omentum by which it was surrounded. I got hold of the feet at length, and brought them down, but was compelled to use very considerable force; as the uterus which was completely ruptured, had no power to aid in expelling the child. As soon as the head reached the superior strait, I found that it could not be made to descend farther, and soon ascertained the cause to be preternatural enlargement, the result of dropsical effusion within the cranium. I immediately passed up a bistoury, and made an incision just above the left ear, the face of the fœtus looking to the symphysis pubis, and thus evacuated a large quantity of water; after which, the head, enormously enlarged by dropsical effusion, passed without difficulty. The placenta was not half its usual size. I had a bandage well adjusted, gave the patient a full dose of morphia, left directions to keep her perfectly quiet, and departed under the impression communicated to her husband, that she would die in the course of the day. She died the next morning.

Polypus of the Uterus. The next case that I shall present, and to me by far the most interesting, is one of Polypus of the Uterus.

The first time I was called to visit this patient, a negro woman about 46 years of age, was in the spring of 1845.

The history that I then received of the case was as follows: About two years previously when it was thought to be about the time her menses ought to cease, she was attacked with violent menorrhagia. The loss of blood was so great, at times, as to threaten immediate death. A skilful physician was called in, who used the ordinary remedies for suppressing this kind of hemorrhage, and she appeared to do pretty well for a short time; but the hemorrhage returned again and again, until she was worn down to a mere skeleton, and all hope of her recovery was lost. For a long time her mistress had not sent for the physician, but kept ready a quantity of acet. plumb. and opium, with which the hemorrhage was easily controlled.

By the advice of my friend and preceptor John R. Frayser, M. D., of Memphis, I applied a blister over the sacrum, and put her upon the use of the infusion of secale cornutum, mineral tonics and a generous diet, and flattered myself for a time that, with these simple means, I should effect a cure; but I soon discovered my error.

Finally, as it had been neglected before, I determined to make an examination to see if possible what was the source of this obstinate hemorrhage. Accordingly in September last proceeding to explore the vagina and os tincæ, Dr. Frayser and myself satisfied ourselves of the existence of a polypus attached to the cervix. The cause was now clear enough. I executed its removal in the following manner:

I used a double silver canula and a waxed thread of a size sufficient to fill accurately the instrument; tying one end of the ligature to one shoulder of the canula, and passing the other end through both barrels, leaving a loop at the other extremity, and two feet of the ligature hanging out, I took the instrument in my left hand and with my right encircled the pedicle of the tumor without much difficulty. Then drawing tightly upon the end of the ligature that hung out with my right hand, while with the left I gave to the point of the instrument the proper direction, to secure the thread around the neck of the tumor as near its attachment as possible, I secured the ligature to the other shoulder of the instrument, and the operation was completed. This was on the first day of October last. I visited the patient the next day and tightened the ligature, and from the extremely offensive smell, I concluded that the circulation in the tumor had been completely cut off. On the next day the instrument, tumor and all came away together, it being only about 48 hours from the time the ligature was applied.

I had the parts well cleansed with a solution of chloride of soda and infusion of chamomile, using them alternately for several days, until the parts ceased to emit an offensive odor. I then put her upon the use of the prussiate of iron, ordered a genorous diet and gentle exercise in the open air, as soon as she was able to walk out. She recovered rapidly, and has menstruated regularly ever since, notwithstanding she says she is 48 years old and has given birth to twelve healthy children, nine of whom are now living; she has perfectly recovered her health, and may yet have several children. A case occurred in Cumberland county, Virginia, where a lady, at fifty-five years of age, gave birth to two fine boys. Other similar cases I believe are on record.

Prof. Dewees in commencing his article upon this disease, says: "This disease of the uterus, the author has never seen, he is therefore under the necessity of drawing his account of the polypus from the experience of others." And again, he remarks: "It would seem to be fairly presumable that this disease is of more rare occurrence in this country than in Europe, since, in the practice of the gentlemen just named, (Levret, Denman, and Clarke) many cases have occurred, while in this, the experience of a number of gentlemen whom I have consulted, has not furnished one."

I am inclined to believe that this disease is of much more frequent occurrence in this country than is generally believed, the opposite opinion obtaining, probably, from the fact that it is not looked for. An examination ought always to be made in obscure cases of uterine disease, since in affections of the kind just described it is clear that nothing but an operation can rescue the patient from the grave.

January 19th, 1847.

